# Metabolomics-Based Screening of Biofilm-Inhibitory Compounds against *Pseudomonas aeruginosa* from Burdock Leaf

**DOI:** 10.3390/molecules200916266

**Published:** 2015-09-08

**Authors:** Zaixiang Lou, Yuxia Tang, Xinyi Song, Hongxin Wang

**Affiliations:** 1 State Key Laboratory of Food Science and Technology, School of Food Science and Technology, Jiangnan University, Wuxi 214122, China; E-Mails: jingxin606@gmail.com (Y.T.); sxysong@ucdavis.edu (X.S.); 2 State Key Laboratory of Dairy Biotechnology, Technology Center of Bright Dairy and Food Company Ltd., Shanghai 200436, China; 3 Department of Food Science and Technology, University of California, Davis, CA 95616, USA

**Keywords:** screening, anti-biofilm compounds, burdock leaf, metabolomics, data analysis

## Abstract

Screening of anti-biofilm compounds from the burdock leaf based on metabolomics is reported here. The crystal violet assay indicated 34% ethanol elution fraction of burdock leaf could completely inhibit biofilm formation of *Pseudomonas aeruginosa* at 1 mg·mL^−1^. Then, the chemical composition of burdock leaf fraction was analyzed by ultra-performance liquid chromatography-mass spectrometry (UPLC-MS) and 11 active compounds (chlorogenic acid, caffeic acid, *p*-coumaric acid, quercetin, ursolic acid, rutin, cynarin, luteolin, crocin, benzoic acid, and Tenacissoside I) were identified. Lastly, UPLC-MS analysis was employed to obtain the metabolic fingerprints of burdock leaf fractions before and after inhibiting the biofilm of *Pseudomonas aeruginosa*. The metabolic fingerprints were transformed to data, analyzed with PLS-DA (partial least squares discriminant analysis) and the peaks whose area was significantly changed were found out. Thus, 81 compounds were screened as potential anti-biofilm ingredients. Among them, rutin, ursolic acid, caffeic acid, *p*-coumaric acid and quercetin were identified and confirmed as the main anti-biofilm compounds in burdock leaf. The study provided basic anti-biofilm profile data for the compounds in burdock leaf, as well as provided a convenient method for fast screening of anti-biofilm compounds from natural plants.

## 1. Introduction

As a unique group life style that grows on solid surface, biofilm has multicellular structures. Biofilm formation is a natural tendency of bacteria [1]. In food industry sectors, such as fresh produce, dairy processing, poultry processing and red meat processing, biofilm is a stubborn source of pollution [2,3]. When the contamination of food occurs, the source of the problem is often biofilm. A great deal of evidence indicates that biofilm mode of life results in growing resistance to antibacterial agents [4,5]. Compared to planktonic cells, biofilms are more resistant to antibacterial products. Searching for new strategies to inhibit biofilm is very urgent [6,7].

In recent years, the efficiency of plant components on inhibition of biofilm has attracted more and more attention [8,9,10]. Honraet [5] found a variety of plant extracts significantly inhibited the biofilm formation of *Staphylococcus aureus*. Figueiredo *et al.* [11] found that *Plectranthus barbatus* and *Plectranthus ecklonii* leaves had anti-biofilm activity on *Streptococcus sobrinus* and *Streptococcus mutans*, with IC_50_ of 0.6–3.1 mg·mL^−1^. However, the material basis for biofilm inhibition or the active ingredients of these plants have not been systemic studied. Furthermore, separation and purification are commonly used to find active ingredients, whereby steps are complex and the functional components are easily lost. Hence, it is urgent to develop a new and selective method for the screening of active ingredients.

Dynamic changes of all small molecule metabolites of biological systems in response to external stimuli can be well analyzed in metabolomics [12,13,14]. Through investigating the changes of metabolome after the treating by drugs or plant components, multidimensional data of the whole research system can be obtained. After statistical analysis, their internal relations could be found, the key biomarkers would be screened, and the effective material basis of plant components could be obtained [15,16,17,18]. Therefore, metabolomics can be employed to comprehensively and accurately screen active compounds, effectively avoiding the complicated separation and purification process as well as the complex process of activity evaluation. In the present paper, a method for screening of anti-biofilm compounds from plants based on metabolomics has been proposed.

In this study, the anti-biofilm activity of burdock (*Arctium lappa*) leaves against *Pseudomonas aeruginosa* was evaluated. Then, UPLC-MS was used to obtain the metabolic fingerprint of burdock leaf fractions before and after inhibiting *Pseudomonas aeruginosa*, and multivariate data analysis was applied to screening potential anti-biofilm ingredients. This method demonstrates the first use of metabolomics analysis as an effective approach for fast screening of anti-biofilm compounds from plants.

## 2. Results and Discussion

### 2.1. Anti-Biofilm Activity of Burdock Leaf Fraction against Pseudomonas aeruginosa

The 34% ethanol elution fraction of burdock leaf significantly inhibited the formation of *Pseudomonas* *aeruginosa* biofilm. When the concentration of burdock leaf fraction was 1 mg·mL^−1^, the formation of biofilm was completely inhibited and the inhibition rate on biofilm formation was 100%. However, when the concentration was less than 1 mg·mL^−1^, it could only partly inhibit biofilm formation. The anti-biofilm activities increased with increasing concentration of burdock leaf fraction.

### 2.2. Chemical Composition of Burdock Leaf Fraction

**Figure 1:**
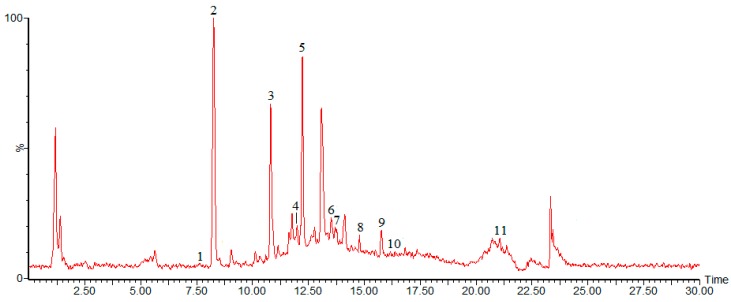
Chromatogram of 34% ethanol elution fraction from burdock leaf in negative ion mode.

The chromatogram of the fraction of burdock leaf is shown in Figure 1. Peak identification was performed by comparing retention times (t_R_), UV-Vis spectra and mass spectra (Table 1) with those of reference standards and literature data.

**Table 1:** Characterization of compounds in the fraction of burdock leaf using ultra-performance liquid chromatography (UPLC) with photo-diode array and electrospray ionization mass spectrometry detection.

Peak	t_R_	M	[M − H]^−^	Fragment Ions	λ_max_	Identification
Peak 1	7.62	354	353	191	326	chlorogenic acid
Peak 2	8.25	180	179	135	324	caffeic acid
Peak 3	10.90	164	163	119	303	*p*-coumaric acid
Peak 4	12.04	122	121	105	222	benzoic acid
Peak 5	12.22	610	609	610	256	rutin
Peak 6	13.67	977	976	487	442	crocin
Peak 7	13.51	516	515	191	295	cynarin
Peak 8	14.82	302	301	151	256,368	quercetin
Peak 9	15.75	286	285	571	210,349	luteolin
Peak 10	16.22	814	813	315	251	Tenacissoside I
Peak 11	21.07	456	455	219	210	ursolic acid

Peak1 was identified as chlorogenic acid with λ_max_ of 326 and t_R_ of 7.62. The [M − H]^−^ peak of 353 (along with the fragment ions at 191) was similar to those of standard chlorogenic acid and those reported in the literature [19]. Peak 2, with the t_R_ of 8.25, was identified as caffeic acid (λ_max_ 324) and the [M − H]^−^ peak was observed at *m*/*z* 179. Its characteristic fragment ions, such as *m*/*z* 135, were also identical with those of the standard and those reported by Tarnawski [20]. Peak 3 exhibiting an [M − H]^−^ ion at *m*/*z* 163 with the λ_max_ of 303 and a t_R_ of 10.90, was identified as *p*-coumaric acid. It also showed the release of predominant fragment ion at *m*/*z* 119. Its characteristics were identical with those of the standard. Compared with the standard benzoic acid, Peak 4 was identified as benzoic acid with the t_R_ of 12.04 and λ_max_ of 222, its [M − H]^−^ peak was observed at *m*/*z* 121. The fifth peak yielding [M − H]^−^ at *m*/*z* 609 was identified as rutin with the t_R_ of 12.22 and λ_max_ of 256. Peak 6, with the t_R_ of 13.51 and λ_max_ of 295, was identified as cynarin and the [M − H]^−^ peak of it was observed at *m*/*z* 515. Its characteristic fragment ions, such as *m*/*z* 191 and 349, were in consistent with those reported in the literature [21,22]. Peak 7, with the tR of 13.67, was identified as crocin (λ_max_ 442) and the [M − H]^−^ peak of crocin was observed at *m*/*z* 976. Its characteristic fragment ions, such as *m*/*z* 487, were also identical with those of the standard and those reported by Kelebek [23]. Peak 8, was identified as quercetin, with the t_R_ of 14.82 and λ_max_ of 256, 368. It also showed the release of predominant fragment ion at *m*/*z* 151, these results were in agreement with those reported in the literature [24]. Peak 9, with the tR of 15.75, was identified as luteolin and the [M − H]^−^ peak was observed at *m*/*z* 285. Peak 10, with the t_R_ of 16.22, was identified as Tenacissoside I (λ_max_ 251) and the [M − H]^−^ peak was observed at *m*/*z* 814. Peak 11 yielded [M − H]^−^ at *m*/*z* 455, with λ_max_ of 210 and t_R_ of 21.07, was identified as ursolic acid. The characteristics of all the compounds identified were found to be identical with those of standard compounds.

### 2.3. Screening of Active Compounds from Burdock Leaf Fraction Based on Metabolomics

#### 2.3.1. UPLC-MS Data Processing and Multivariate Data Analysis

A data array composed of the variables of samples, retention time t_R_, *m*/*z* values (molecular features), and normalized signal intensity of the *m*/*z* value was generated after UPLC-MS data were processed by MarkerLynx software (4.1; WATERS: Manchester, UK) [25]. Subsequently, checked *m*/*z* values and those being shown in the blank samples considered as noise or contaminants were moved. In order to treat the missing values, frequency of occurrence of mass ion (nonzero value) less than 80% was removed [26]. Then unit variance method was used for preprocessing of the data. In order to find significant differences between groups, the resulting data arrays were used afterwards for multivariate statistical analysis, which were principal component analysis (PCA) and Partial least squares discriminant analysis (PLS-DA) with SIMCAP + 11 software package [27]. Cross-validation was conducted to verify whether PLS-DA was a good model. The variable importance in projection (VIP) was used to discern the differences. The VIP value of each variable was got from PLS-DA score plot, which was used for classification purposes in metabolomics studies, and to indicate its contribution to the classification of samples. When the VIP value is higher, the metabolites are considered important for discriminating between different groups. The metabolites with a larger VIP value represented higher contribution to the discrimination between two groups. According to the VIP values and other results of data analysis, screened the peaks with significant decrease of peak area. The selected components were the compounds with biofilm inhibition potential. Then further identified the compounds corresponding to the peaks and find out the active compounds with anti-biofilm activity.

#### 2.3.2. Screening Potential Anti-Biofilm Compounds

**Figure 2:**
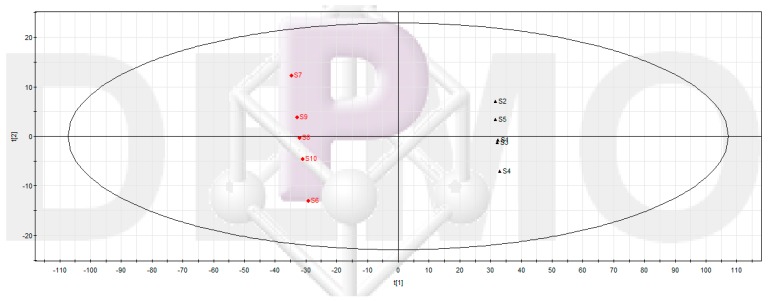
Partial least squares discriminant analysis (PLS-DA) scores plot of Groups 2 and 3. The main composition of the second and the third group of samples could be considered as the composition of burdock leaf fraction before and after inhibiting *Pseudomonas aeruginosa* biofilm, respectively. Group 2 includes samples 1–5 (S1–S5) and Group 3 includes samples 6–10 (S6–S10). R^2^X = 0.77, R^2^Y = 0.99, Q^2^ = 0.99.

The results of UPLC-MS chromatograms of samples in Groups 2 and 3 were converted into a data array. After data processing and multivariate data analysis, the data were displayed as PLS-DA score plots (Figure 2). As shown in Figure 2, the data of Groups 2 and 3 were clearly clustered into two groups. This meant Groups 2 and 3 were indeed different in the levels or occurrences of their components.

There were area reductions in peaks of burdock leaf fractions after inhibiting *Pseudomonas* *aeruginosa*, which was probably because some compounds in burdock leaf fractions had exerted inhibition effect on biofilm. The compounds that had greatest impact on the division of the two sample groups were found, so as to find the compounds whose peak area reduced significantly. Thus, PCA and PLS-DA were performed to find the components contributing the most to the difference between these two groups of samples. The sevenfold cross-validation results had the highest accuracy. All samples could be put in 95% confidence intervals. The peaks with VIP value greater than 1.15 were obtained. Among these peaks, only the peaks with reducing area were selected. Then, we further conducted nonparametric test using SPSS 17.0, and *p* < 0.05 was considered to indicate statistical significance. Thus, 81 ion t_R_-*m*/*z* pair points with significant reduction of peak area were screened out (Table S1), and the 81 compounds corresponding to these selected ions (peaks) were potential ingredients with anti-biofilm activity. The results of these components provided a clear direction for the screening of active compounds with anti-biofilm activity, narrowing the scope of screening. Meanwhile, the results provided technical guidance and basic data for future separation of these anti-biofilm compounds from burdock leaf.

According to relevant information obtained in Section 3.2, similarly, some peaks listed in Table S1 were identified. As shown in Table 2, No. 6 was identified as caffeic acid with the t_R_ of 8.25. No. 25 was identified as rutin with the t_R_ of 12.22. No. 60, No. 65, No. 73, No. 79 and No. 81 were identified as cynarin, crocin, *p*-coumaric acid, quercetin and ursolic acid, respectively. Meanwhile, combing the database (HMDB, METLIN, Massbank, *etc.*) as references, other potential anti-biofilm compounds are being identified on the basis of relevant literature, database and the fragmentation patterns of mass spectrometry. Referring to the activity evaluation experiments, rutin, ursolic acid, caffeic acid, *p*-coumaric acid and quercetin were found to exhibit significant biofilm inhibitory activity. As shown in Table 2, the lowest concentrations of rutin, ursolic acid, caffeic acid, *p*-coumaric acid and quercetin that could completely inhibit (with inhibition rate of 100%) the formation of biofilm of *Pseudomonas aeruginosa* were 0.5, 0.5, 0.5, 0.25 and 0.5 mg·mL^−1^, respectively. Thus, these five compounds were the main anti-biofilm ingredients in burdock leaf.

**Table 2:** Biofilm inhibition activity of the compounds identified among the potential anti-biofilm ingredients.

Number	Retention Time	Compound	LCB (mg·mL^−1^)
6	8.2526	caffeic acid,	0.5
25	12.2554	rutin	0.5
60	13.5154	cynarin	>4
65	13.6679	crocin	>4
73	10.9081	*p*-coumaric acid	0.25
79	14.8257	quercetin	0.5
81	21.0689	ursolic acid	0.5

LCB: the lowest concentrations of the compound that could completely inhibit (with inhibition rate of 100%) the formation of biofilm of *Pseudomonas aeruginosa*.

Through analyzing the changes of burdock leaf fraction before and after biofilm inhibiting, following a series of data statistics and analysis, as well as structural identification, a series of potential active compounds with biofilm inhibiting activity could be screened out from burdock leaves. Compared to the conventional methods for active compounds screening, this approach eliminated or simplified the process of gradual separation, purification and the repeated activity evaluation in traditional screening methods. It greatly simplified the screening process of active ingredients, saved a lot of manpower and material resources, which was in favor of significantly accelerating the screening efficiency for active ingredients. The proposed approach in this study might provide a new way for fast and convenient screening of biofilm inhibition components from plants.

Next, this screening method will be applied to other plants for screening of anti-biofilm ingredients, in order to further improve this method, providing the basis for wide applications of the proposed approach. The synergy effect between these screened anti-biofilm compounds will also be investigated.

## 3. Experimental Section

### 3.1. Chemicals and Reagents

Chlorogenic acid (98%), caffeic acid (98%), *p*-coumaric acid (99%), quercetin (97%), ursolic acid (98%), rutin (98%), luteolin (98%), crocin (98%), cynarin (97%), benzoic acid (99%) and Tenacissoside I (97%) were purchased from Aladdin (Shanghai, China) and Sigma (Shanghai, China). Burdock leaf was provided by Xuzhou Wangda Farm and Sideline Products Co., Ltd. (Xuzhou, China).

### 3.2. Bacteria Culture

The strain *Pseudomonas aeruginosa* ATCC 9027 was purchased from Guangdong Microbiological Culture Collection Center (Guangzhou, China). It was activated and inoculated into fresh LB broth, then cultivated at 37 °C for 12 h. A bacterial suspension of about 108 CFU·mL^−1^ was prepared for the following experiments.

The main composition of the second and the third group of samples could be considered as the composition of burdock leaf fraction before and after inhibiting *Pseudomonas aeruginosa* biofilm, respectively. Group 2 includes samples 1–5 (S1–S5) and Group 3 includes samples 6–10 (S6–S10).

### 3.3. Burdock Leaf Fraction Preparation

Dry burdock leaf was pulverized into powder. The powder of burdock leaf was extracted in a flask containing 30% ethanol solution; the ratio of solvent to solid was 20:1 (mL·g^−1^). The extraction was carried out at 30 °C, 250 rpm for 10 h. The extracts were filtered and then concentrated using a rotary evaporator at 50 °C under vacuum. The crude extracts were dissolved in water, then loaded into a glass column (6 cm × 100 cm) filled with macroporous resin (HPD-100, Cangzhou Bon Adsorber Technology Co., Ltd., Cangzhou, China). In the macroporous resin column chromatography, 20%, 34% and 70% ethanol solutions were used to desorb targeted components at a successively flow of 1.5 BV·h^−1^, respectively. Only the 34% ethanol elution fraction was collected and dried. Then, the 34% ethanol solution elution fraction was obtained and used for the following experiments.

### 3.4. Samples Preparation

Standard samples (chlorogenic acid, caffeic acid, *p*-coumaric acid, quercetin, ursolic acid, rutin, luteolin, crocin, benzoic acid and Tenacissoside I) were dissolved with methanol (HPLC grade); the concentration of each standard was 1 mg·mL^−1^. All of the standard samples were stored at 4 °C before use.

The samples preparation methods and sample groups are shown in Figure S1. The first group of samples was the solution of 34% ethanol elution fraction of burdock leaf (2 mg·mL^−1^). As shown in Figure S1, one milliliter of this burdock leaf fraction solution (2 mg·mL^−1^) was added to tubes containing 2.9 mL of LB broth and 0.1 mL of bacteria suspension (108 cfu·mL^−1^). Then the mixture was incubated at 37 °C for 24 h and centrifuged at 7000 r·min^−1^ for 5 min, and the supernatant obtained was named the third group of samples. The second group of samples was prepared by the same method as the third group without inoculation of bacteria. Each group had five samples. The main composition of the second and the third group of samples could be considered to be the composition of burdock leaf fraction before and after inhibiting *Pseudomonas aeruginosa* biofilm, respectively. Acetonitrile precipitation [28] was applied to remove proteins before UPLC-MS analysis. Three times volume of acetonitrile was added to the third and the second group of samples, standing for 10 min, and then centrifuged for 1 min at 12,000 r·min^−1^. The solution obtained was transferred to EP tubes for use.

### 3.5. Anti-Biofilm Activity

Anti-biofilm activity of the fraction of burdock leaf was evaluated by the method of crystal violet staining [29]. The solution of 34% ethanol elution fraction of burdock leaf (100 μL), which had serial dilutions, were added to the wells of a 24-well culture plate (Sarstedt, Newton, NC, USA) containing 800 μL of fresh broth medium and 100 μL bacteria suspension (108 cfu·mL^−1^). After incubation for 24 h, growth medium was removed. The contents of the wells were rinsed 3 times with PBS, fixed by drying for 3 h in a 37 °C incubator. Once the wells were fully dried, 1 mL of 0.1% crystal violet stain was added to wells to stain for 15 min. The excess stain was rinsed off with tap water and then 1 mL of 95% (*v*/*v*) ethanol was added to each well for 1 h to release the stain. One hundred microliters from each well was then transferred to a new plate for spectrophotometric analysis (Optical Density (OD) 570 nm). For all the assays, a positive control without burdock leaf fraction and a negative control without inoculation were prepared. The procedure was performed in triplicate and the means were calculated. The anti-biofilm activity of rutin, ursolic acid, caffeic acid, p-coumaric acid, crocin, cynarin and quercetin was also evaluated.

### 3.6. Chemical Composition Analysis and Anti-Biofilm Compounds Screening

The chemical composition of the 34% ethanol elution fraction of burdock leaf was investigated by ultra-performance liquid chromatography-tandem mass spectrometry (UPLC-MS). High resolution mass was measured for metabolites after UPLC separation. Chromatographic separations were performed on an Acquity UPLC system (Waters, Milford, MA, USA) equipped with BEH C18 column (50 × 2.1 mm, 7 μm, Waters, Milford, MA, USA), applying the following binary gradient at a flow rate of 0.3 mL·min^−1^: 0–1 min, isocratic 95% A (water/formic acid, 99.9/0.1 (*v*/*v*)), 5% B (acetonitrile/formic acid, 99.9/0.1 (*v*/*v*)); 1–16 min, linear from 5% to 95% B; 16–18 min, isocratic 95% B; 18–20 min, isocratic 5% B. The injection volume was 2 μL.

The eluted output from the UPLC equipment was directly connected to a SYNAPT Mass Spectrometer (Waters, Milford, MA, USA), equipped with an electrospray ionization source operating in negative mode, using the following instrument settings: nebulizer gas, nitrogen, capillary, 3000 V, Cone voltage 20 V, desolvation temperature 400 °C, ion source temperature 100 °C, desolvation gas flow 500 L·h^−1^, cone gas flow 50 L·h^−1^.

Standard samples and the first group of samples were used for chemical composition analysis of burdock leaf fraction. The peak belongs to each compound was determined by using the software Masslynx (Waters, Milford, MA, USA). Identification of the compounds from burdock leaf was achieved by comparison of retention times, MS and UV spectra with those of standards and the literature. The second and the third group of samples were used for screening of anti-biofilm compounds in burdock leaf based on metabolomics.

## 4. Conclusions

The 34% ethanol elution fraction of burdock leaf significantly inhibited the biofilm formation of *Pseudomonas aeruginosa*. Chlorogenic acid, caffeic acid, *p*-coumaric acid, quercetin, ursolic acid, rutin, cynarin, luteolin, crocin, benzoic acid and Tenacissoside I were identified in the fraction through UPLC-MS analysis. Through analyzing the metabolomics changes of burdock leaf fraction before and after inhibiting biofilm, coupled with UPLC-MS data processing and multivariate data analysis, 81 variables were screened as potential anti-biofilm ingredients; among them, rutin, ursolic acid, caffeic acid, *p*-coumaric acid and quercetin were identified and confirmed as the main anti-biofilm compounds in burdock leaf fraction. The proposed method simplified the complex purification and activity evaluation process of the traditional method for screening of active ingredients, and it is expected to become a convenient and practical method for fast screening of the active ingredients from plants.

## Supplementary Materials

Supplementary materials can be accessed at: http://www.mdpi.com/1420-3049/20/09/16266/s1.
