# Supplementary material for: Metabolomics-Based Screening of Biofilm-Inhibitory Compounds against Pseudomonas aeruginosa from Burdock Leaf
Source: Molecules. 2015 Sep 8;20(9):16266–77. doi: 10.3390/molecules200916266 (PMC6331861; doi:10.3390/molecules200916266)
Supplement: Supplementary file 1 [file molecules-20-16266-s001.pdf]

Supplementary Materials

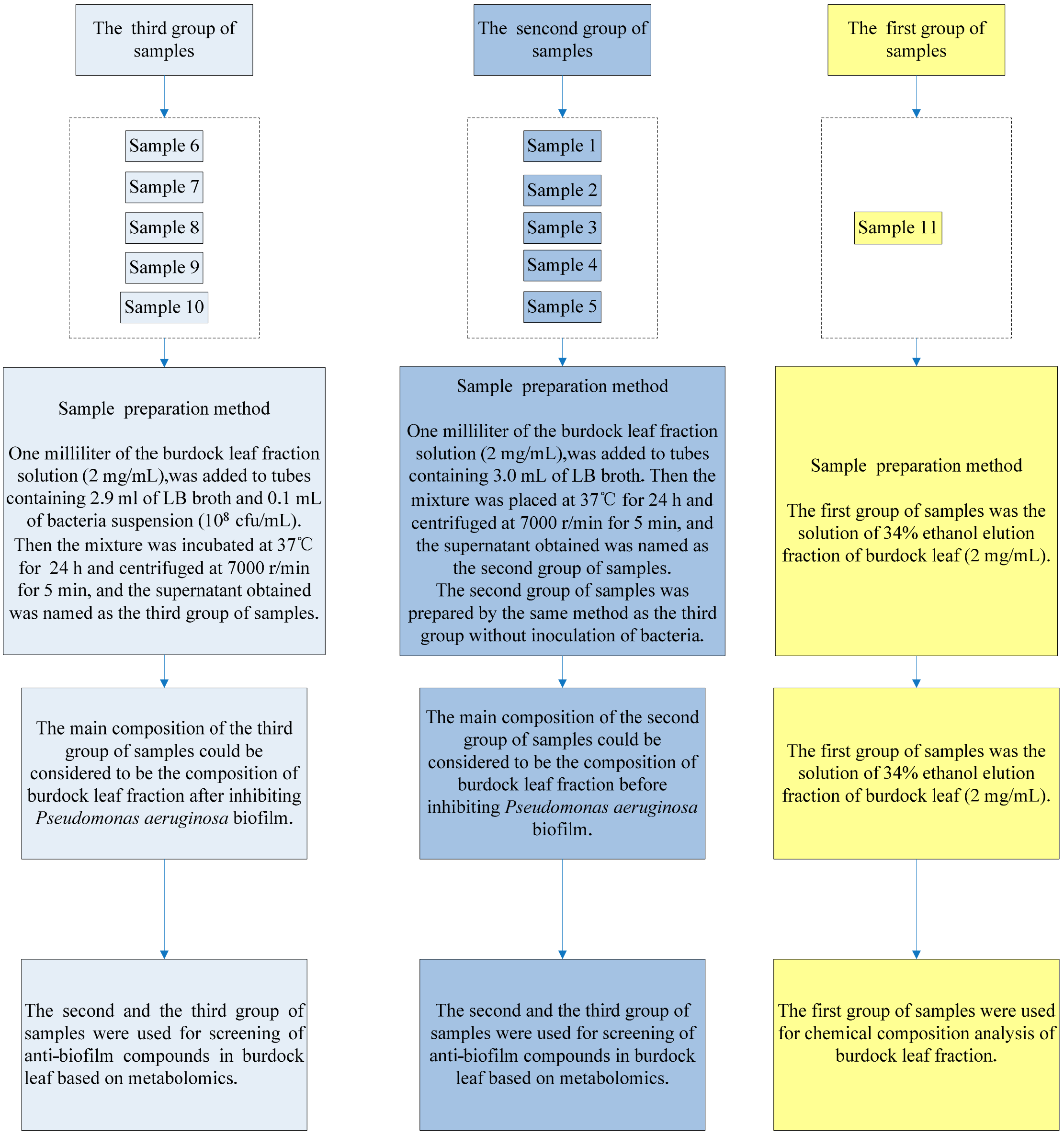

Figure S1. Samples preparation methods and sample groups.

Table S1. Peaks (ion t_R–m/z pair points) with significant reduction of area.

Number	Retention Time	Mass	APACA2	APACA3	VIP
1	10.7922	431.1973	149.3706	48.09534	1.16476
2	12.8307	345.1053	146.011	46.95214	1.16517
3	13.7507	187.1016	83.8051	20.65404	1.16598
4	13.0333	462.2222	61.2066	2.861	1.16657
5	12.1489	331.1315	65.777	17.88796	1.16365
6	8.2526	179.04	58.2216	12.61814	1.16438
7	12.6807	343.1146	57.6269	15.65608	1.15796
8	13.0522	515.1301	50.3402	5.81968	1.16292
9	13.219	449.1603	51.3319	12.7476	1.16711
10	10.7027	341.0997	50.73805	11.86934	1.16689
11	12.6042	449.1584	50.54455	13.48902	1.1626
12	13.4485	161.0638	48.365	9.75022	1.16484
13	10.4155	378.1972	46.1295	8.11206	1.16495
14	10.9224	465.1537	48.20445	12.25212	1.1591
15	12.6271	493.1866	47.05215	12.87938	1.16302
16	13.7375	125.1017	43.0998	10.24674	1.16314
17	13.8431	461.2509	43.588	13.30158	1.16271
18	12.2932	333.1465	42.2346	11.66186	1.16083
19	10.6824	345.1129	40.0473	10.5863	1.1629
20	10.7982	421.1728	39.82695	12.68882	1.15229
21	14.1265	477.1913	38.40615	9.9285	1.16309
22	10.4134	114.0594	36.38575	7.80678	1.16376
23	10.7658	385.1962	37.10785	10.64166	1.16366
24	13.2863	451.1743	35.204	9.29926	1.16157
25	12.2254	609.1527	33.7945	7.04942	1.16236
26	11.4927	461.2515	34.6727	9.83132	1.16285
27	12.8332	379.1497	29.5326	1.80134	1.16529
28	13.043	114.0595	28.92435	1.59552	1.16435
29	13.8714	243.1289	30.87175	5.441	1.16091
30	10.6735	323.1325	32.9918	10.11388	1.15403
31	11.9171	465.152	31.2695	7.9291	1.1526
32	13.0533	353.0954	27.4747	2.19184	1.15546
33	12.6058	427.2063	32.21225	10.5928	1.16434
34	14.5251	491.1675	28.89495	6.1879	1.15801
35	10.8742	281.1435	29.56155	8.09338	1.16617
36	11.603	449.2522	29.118	8.29244	1.16556
37	13.4472	205.0572	26.8918	4.4431	1.16363
38	11.7734	431.2009	28.9007	8.1828	1.16193
39	11.0986	495.202	27.91205	7.17914	1.15157
40	13.3638	333.1464	27.2168	6.94594	1.16145
41	12.5955	473.2154	27.86445	8.80852	1.16056

Table S1. *Cont.*

Number	Retention Time	Mass	APACA2	APACA3	VIP
42	11.4214	319.1276	25.11335	5.93506	1.155
43	10.3068	363.087	24.49825	5.52614	1.15936
44	14.1176	973.5215	22.8987	4.7582	1.16324
45	14.4314	243.1283	20.1257	4.07526	1.15807
46	13.5119	231.1266	18.4781	2.6985	1.16065
47	11.0125	473.2161	19.3952	5.16984	1.16118
48	13.4496	117.0752	18.591	3.7141	1.16652
49	12.223	495.2052	18.62465	4.98698	1.16556
50	10.8356	243.18	16.40205	1.77508	1.15695
51	13.4626	461.2476	17.2381	4.78772	1.15959
52	10.628	177.0602	15.17925	3.04924	1.16534
53	13.8488	333.1483	14.56275	2.67206	1.16017
54	14.2355	431.208	13.2235	0.84918	1.15406
55	3.4633	187.1128	16.39245	6.66522	1.15678
56	12.7324	333.1484	14.16585	2.56634	1.15103
57	9.2448	177.0597	13.62345	2.73928	1.16288
58	12.486	413.1626	14.0164	3.7563	1.15809
59	12.2016	431.2017	12.50935	3.41674	1.15955
60	13.5154	515.133	12.1929	3.52052	1.16232
61	11.6032	167.0271	10.49265	0.5055	1.15523
62	12.8928	593.1629	11.79985	2.75446	1.16491
63	11.9434	461.2498	11.98195	3.61658	1.15584
64	10.8005	225.0674	11.98195	3.68714	1.1641
65	13.6679	973.5202	11.10355	2.57788	1.14869
66	14.2177	423.1756	10.89925	2.08334	1.16725
67	13.2068	405.1854	10.792	2.16862	1.16124
68	10.7888	499.1982	10.4078	1.998	1.16465
69	13.8146	151.0067	11.08145	3.60096	1.15298
70	11.4468	401.1962	10.2198	2.5611	1.157
71	12.6073	463.1854	10.6113	3.32776	1.16295
72	12.8344	465.1562	10.6966	3.59636	1.16249
73	10.9081	119.0509	10.0748	2.57582	1.1649
74	11.258	431.1647	9.25265	1.67326	1.15308
75	13.6752	227.1329	9.16445	1.7221	1.16355
76	12.9326	505.1444	9.43115	2.70704	1.15159
77	14.0139	225.1202	9.2104	2.25728	1.16666
78	13.3167	287.0284	8.0005	1.6131	1.15908
79	14.8257	301.0433	7.28355	2.40472	1.15783
80	10.4912	465.1559	6.8645	1.90422	1.1641
81	21.0689	455.3725	3.5295	0.54688	1.1502

These peaks represented the compounds with anti-biofilm activity potential. APACA2: Average peak area of compounds in sample Group 2. APACA3: Average peak area of compounds in sample Group 3. VIP: Variable importance in projection.
